# Thoracic spinal cord cavernous angioma: a case report and review of the literature

**DOI:** 10.1186/1752-1947-8-271

**Published:** 2014-08-08

**Authors:** Giovanni Grasso, Concetta Alafaci, Francesca Granata, Mariano Cutugno, Francesco Maria Salpietro, Francesco Tomasello

**Affiliations:** 1 Neurosurgical Clinic, Department of Experimental Biomedicine and Clinical Neurosciences, University of Palermo, School of Medicine, Via del Vespro 129, Palermo 90100, Italy; 2 Department of Neurosurgery, University of Messina, Via Consolare Pompea, 1, Messina 98126, Italy; 3 Department of Radiological Sciences, University of Messina, Via Consolare Pompea, 1, Messina 98126, Italy

**Keywords:** Cavernous angioma, Intramedullary, Spinal cord

## Abstract

**Introduction:**

Cavernous angiomas of the spinal cord are rare vascular malformations, which account for approximately 5 to 12 percent of spinal cord vascular lesions. They usually originate in the vertebrae, with occasional extension into the extradural space, and intramedullary cavernomas, even if reported in the literature, are very rare.

**Case presentation:**

We report the case of a 34-year-old Caucasian woman affected by a thoracic intramedullary cavernous angioma. Our patient complained of 10-day history of acute dorsal pain, progressive weakness of both lower extremities, worse on the right side, a ‘pins and needles’ sensation in the abdominal region and bladder dysfunction. Magnetic resonance imaging showed, at D5 level, a vascular malformation, which was not documented at spinal angiography. Our patient underwent surgical treatment with total removal of the lesion and her symptoms gradually improved. A histological examination revealed the typical features of a cavernous angioma.

**Conclusions:**

Intramedullary cavernous angioma is a rare lesion that should be diagnosed early and surgically treated before rebleeding or enlargement of the lesion can occur.

## Introduction

Cavernous angiomas are uncommon vascular malformations of the central nervous system (CNS) characterized by abnormally dilated blood vessels, lined by a thin endothelium without intervening normal nervous tissue. They are more common in women and tend to become clinically symptomatic during the third and the fourth decade of life [1]. In the spine, cavernous angiomas represent 5 to 12 percent of spinal cord vascular lesions and they frequently originate in the vertebrae with occasional extension into the extradural space [1]. Intramedullary cavernomas are very rare. They are usually solitary although may be associated with cavernous angiomas in other organs or in the central nervous system [2]. The most common clinical presentation is represented by a slowly progressive myelopathy, but subarachnoid hemorrhage [3] and hematomyelia [4] have also been described.

Since the advent of magnetic resonance imaging (MRI), large series of these relatively rare lesions have been reported with increasing frequency, allowing a better understanding about epidemiology, natural history, clinical presentation, and surgical results [5-12].

Here, the authors describe the clinical, neuroradiological, and surgical features of a new case of intramedullary cavernous angioma in the thoracic spinal cord.

## Case presentation

**Figure 1:**
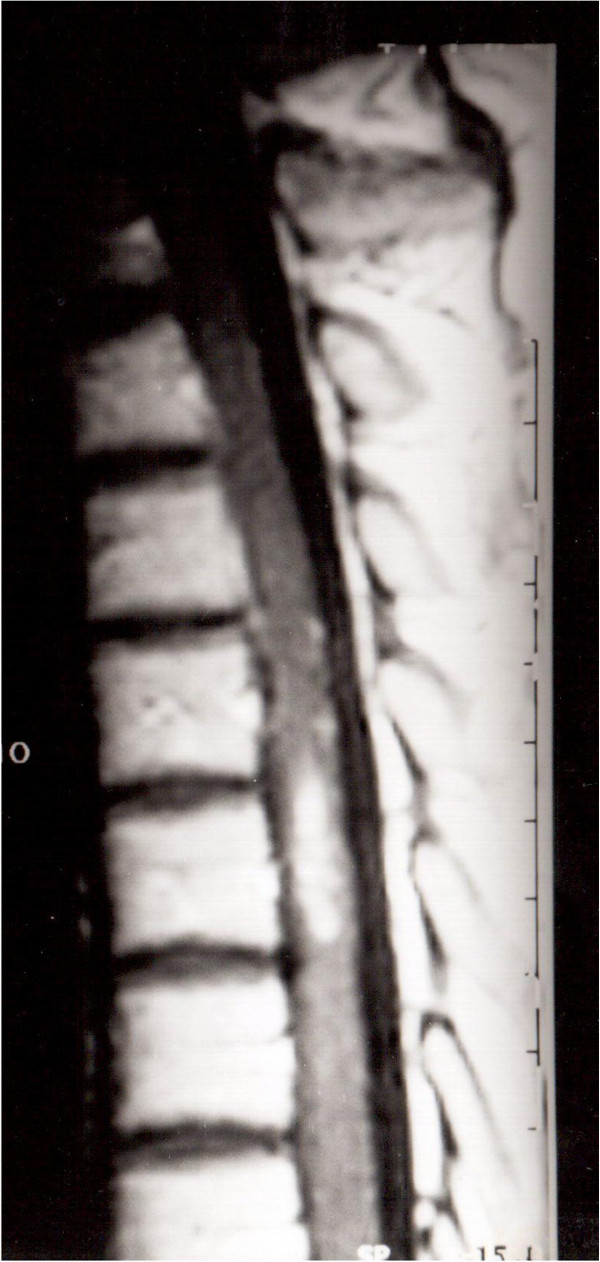
T1-weighted sagittal magnetic resonance image showing a high signal intensity mass at D5 level.

**Figure 2:**
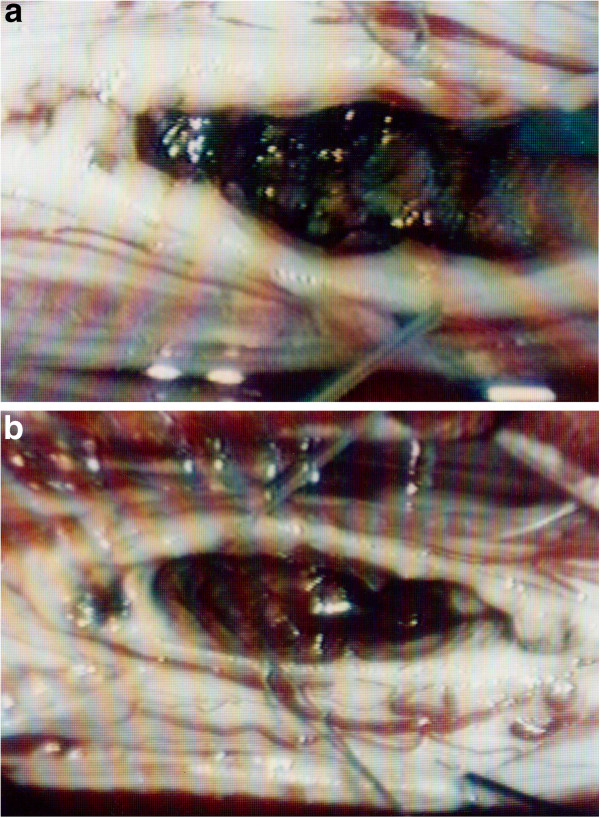
Intraoperative photograph revealing an intramedullary lesion after myelotomy (a); the lesion was carefully dissected out and removed in one piece (b).

**Figure 3:**
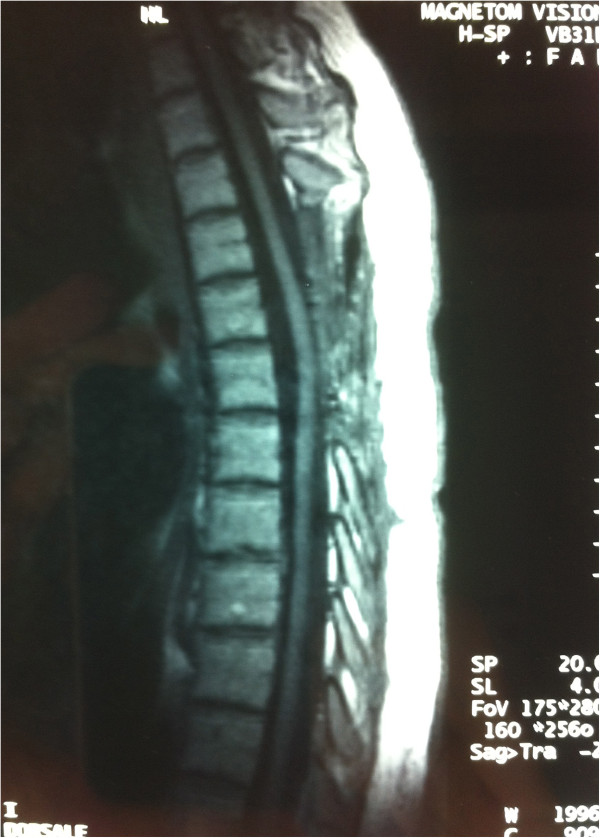
T1-weighted sagittal magnetic resonance image one month later, showing no evidence of residual lesion.

A 34-year-old Caucasian woman was admitted complaining of a 10-day history of acute thoracic pain, progressive weakness of both lower extremities, worse on the right side, a ‘pins and needles’ sensation in the abdominal region, and bladder dysfunction. A neurological examination revealed spastic paraparesis, symmetrically decreased lower-extremity reflexes in her legs, a bilateral positive Babinski sign and decreased pinprick sensation below D6 level.Our patient underwent an MRI scan of the spine, which revealed an intramedullary lesion at D5 level with high-intensity signal relative to the spinal cord on T1-weighted images, spindle-like shaped and 1.5cm in diameter (Figure 1). An MRI scan with angiographic sequences and selective spinal angiography did not show vascular blush or abnormal vascularity.Our patient underwent D4-D5-D6 laminectomy. At the operation, the dura appeared intact. Once exposed, the spinal cord appeared normal on the surface although it was swollen at D5 level. A midline cordotomy was performed and a well-circumscribed dark-bluish lesion, measuring 1.5×0.5cm in diameter, was revealed deep within the spinal cord (Figure 2a). The lesion was carefully dissected out and totally removed in one piece (Figure 2b). A histological examination revealed the typical features of a cavernous angioma.The immediate postoperative course was uneventful. The paraparesis and sensory deficits gradually improved and our patient was discharged on the 18th postoperative day. A one-month follow-up spine MRI scan revealed no residual lesion (Figure 3). One-year post-operatively, our patient was able to walk again.

## Discussion

Cavernous angiomas are angiographically occult vascular malformations of the central nervous system characterized by closely, abnormal, dilated thin-walled sinusoidal spaces, lined by a single layer of endothelium and lacking in intervening nervous tissue [13]. These vascular malformations are usually slow-growing lesions and they may enlarge in different ways. The most common mechanism of growth is represented by small bleedings, often clinically silent, which create a small cavity around the blood vessels, causing hemosiderin deposition in the surrounding neural tissue. Another possibility is that the nidus may grow over time, increasing the mass of the malformation. Finally, the lesion may undergo acute massive hemorrhage with rapid expansion and acute mass effect. These three possibilities may likely coexist in the mechanism of growth [13]. A possible role in the development of such lesions by venous malformations, which sometimes are associated with cavernous angioma, has been pointed out [14]. One of the most representative series reported in the literature showed the coexistence of cavernous angiomas with cryptic venous anomalies in 16 out of 17 cases of intramedullary cavernoma [15].

In the spine, cavernomas account for 5 to 12 percent of all spinal vascular anomalies, and they usually arise within the vertebral bodies with occasional extension into the extradural space [1]. Recently, 27 series with 352 patients were reviewed to elucidate the epidemiological, natural history, and surgical results [16]. Accordingly, one-half of patients present with progressive deficits while others present acutely or with recurrent symptomatology. Like cerebral cavernous malformations, spinal cord cavernomas tend to become clinically apparent during the third and fourth decades of life and women are slightly more affected. Reports of familial incidence and multiple localizations, involving both CNS and other organs, suggest the possibility of genetic mechanisms in the development of such lesions [17].

Spinal intramedullary cavernous angiomas may be asymptomatic, accidentally found at autopsy in patients with multiple cavernous angiomas, or may have variable patterns of clinical presentation. Progressive neurological deterioration can be confused with demyelinating pathologies, myelitis, intramedullary tumors, and spinal arteriovenous vascular malformation [16]. Subarachnoid hemorrhage [3] and hematomyelia [4] have been reported as initial clinical presentation although the most frequent mode of presentation is progressive myelopathy. The latter is usually caused by progressive enlargement and bleeding of the vascular malformation [1]. It has been suggested that hemosiderin, which may have a neurotoxic effect, and alteration of the surrounding microcirculation may play a role in progression of symptoms in patients whose neuroradiological investigations do not show evident signs of enlargement or bleeding of the cavernous angioma. The appropriate treatment of spinal intramedullary cavernous angiomas depends on understanding the natural history of this condition, including the annual rate of hemorrhage and the clinical outcomes of treatments. It has been shown that the annual rates of hemorrhage of intramedullary spinal cord cavernous angioma range from 1.7 to 4.5 percent [7,16]. However, the main information comes from surgical series, which include most patients with more serious conditions. Therefore, the rate of annual hemorrhage may be higher than for conservatively managed patients. Furthermore, the period of time between the onset of symptoms and diagnosis depends upon the pattern of presentation. Our patient developed acute dorsal pain and paraparesis due to an intramedullary spinal cord hemorrhage. The time between the first symptom and diagnosis was about 10 days. An MRI scan, performed upon admission, revealed the presence of an intramedullary lesion with the neuroradiological features of a cavernoma.

Early surgery with total removal of the malformation should be the goal when approaching these spinal lesions, before enlargement or rebleeding can occur. Intraoperative somatosensory and motor-evoked potential monitoring is used. A subtotal resection should be considered when neurological function is at risk, since rebleeding of the residual lesion can occur.

At surgery, an area of discoloration, swelling or presence of anomalous vascular reticule may often be observed on the surface of the spinal cord. A myelotomy performed over these anomalies usually allows the identification of the lesion within the spinal cord. In our case, a area of swelling was detected over the dorsal surface of the thoracic spinal cord at D5-D6 level. A midline cordotomy revealed a well-circumscribed dark-bluish lesion deep within the spinal cord. The lesion was carefully dissected out and removed in one piece.

A follow-up MRI examination is required to confirm complete removal of the cavernous angioma.

## Conclusions

Intramedullary cavernous angiomas are rare lesions. Early diagnosis and proper treatment can prevent bleeding and dangerous enlargement of the lesion.

## Consent

Written informed consent was obtained from the patient for publication of this case report and any accompanying images. A copy of the written consent is available for review by the Editor-in-Chief of this journal.

## Competing interests

The authors declare that they have no competing interests.

## Authors’ contributions

GG prepared and drafted the manuscript and performed the literature research. CA edited the manuscript and performed the literature research. FG edited the manuscript and performed the literature research. MC edited the manuscript. FMS edited the manuscript. FT performed the operation and was involved in the editing of the manuscript. All authors read and approved the final manuscript.
